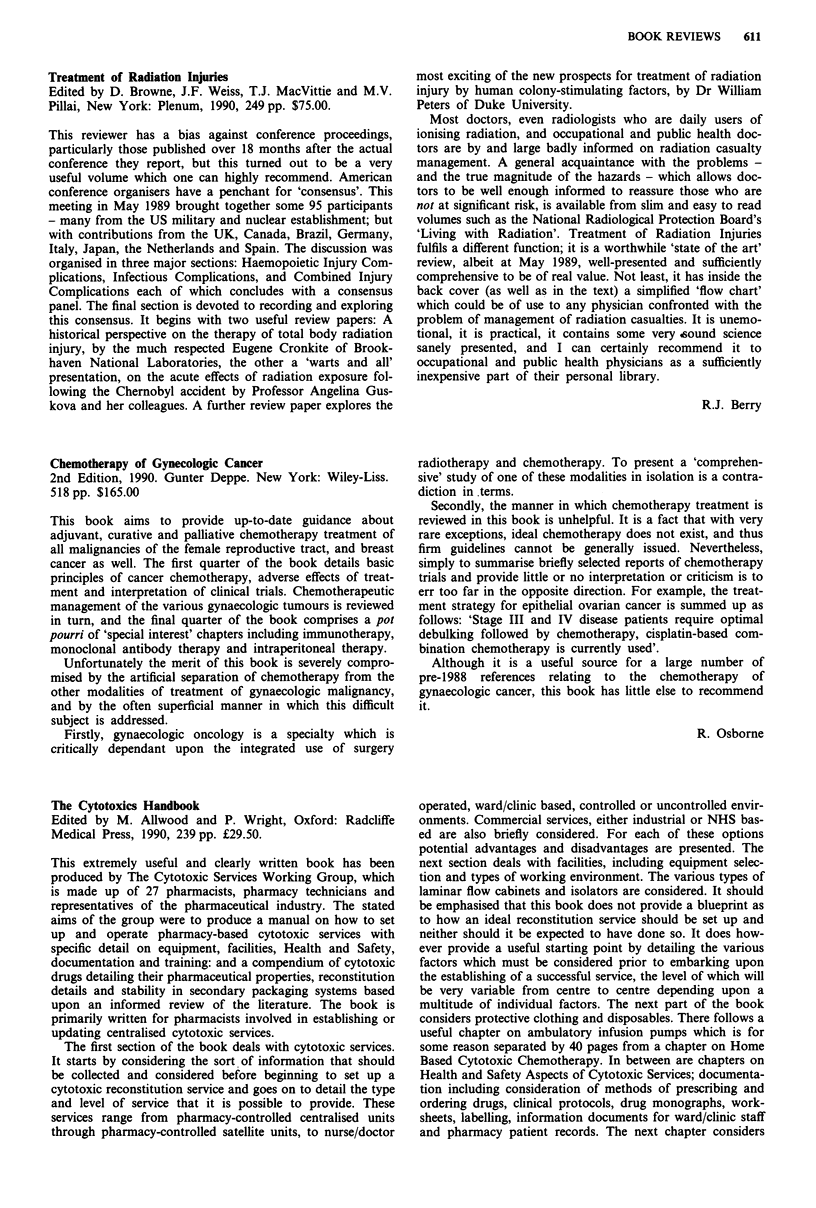# Chemotherapy of Gynecologic Cancer

**Published:** 1991-09

**Authors:** R. Osborne


**Chemotherapy of Gynecologic Cancer**
2nd Edition, 1990. Gunter Deppe. New York: Wiley-Liss. 518 pp. $165.00

This book aims to provide up-to-date guidance about adjuvant, curative and palliative chemotherapy treatment of all malignancies of the female reproductive tract, and breast cancer as well. The first quarter of the book details basic principles of cancer chemotherapy, adverse effects of treatment and interpretation of clinical trials. Chemotherapeutic management of the various gynaecologic tumours is reviewed in turn, and the final quarter of the book comprises a *pot pourri* of 'special interest' chapters including immunotherapy, monoclonal antibody therapy and intraperitoneal therapy.

Unfortunately the merit of this book is severely compromised by the artificial separation of chemotherapy from the other modalities of treatment of gynaecologic malignancy, and by the often superficial manner in which this difficult subject is addressed.

Firstly, gynaecologic oncology is a specialty which is critically dependant upon the integrated use of surgery radiotherapy and chemotherapy. To present a 'comprehensive' study of one of these modalities in isolation is a contradiction in terms.

Secondly, the manner in which chemotherapy treatment is reviewed in this book is unhelpful. It is a fact that with very rare exceptions, ideal chemotherapy does not exist, and thus firm guidelines cannot be generally issued. Nevertheless, simply to summarise briefly selected reports of chemotherapy trials and provide little or no interpretation or criticism is to err too far in the opposite direction. For example, the treatment strategy for epithelial ovarian cancer is summed up as follows: 'Stage III and IV disease patients require optimal debulking followed by chemotherapy, cisplatin-based combination chemotherapy is currently used'.

Although it is a useful source for a large number of pre-1988 references relating to the chemotherapy of gynaecologic cancer, this book has little else to recommend it.

R. Osborne